# NTFD—a stand-alone application for the non-targeted detection of stable isotope-labeled compounds in GC/MS data

**DOI:** 10.1093/bioinformatics/btt119

**Published:** 2013-03-11

**Authors:** Karsten Hiller, André Wegner, Daniel Weindl, Thekla Cordes, Christian M. Metallo, Joanne K. Kelleher, Gregory Stephanopoulos

**Affiliations:** ^1^Luxembourg Centre for Systems Biomedicine, University of Luxembourg, L-4362 Esch-Belval, Luxembourg, ^2^Department of Bioengineering, University of California, San Diego, La Jolla, CA 92093, USA and ^3^Department of Chemical Engineering, Massachusetts Institute of Technology, Cambridge, MA 02140, USA

## Abstract

**Summary:** Most current stable isotope-based methodologies are targeted and focus only on the well-described aspects of metabolic networks. Here, we present NTFD (non-targeted tracer fate detection), a software for the non-targeted analysis of all detectable compounds derived from a stable isotope-labeled tracer present in a GC/MS dataset. In contrast to traditional metabolic flux analysis approaches, NTFD does not depend on any *a priori* knowledge or library information. To obtain dynamic information on metabolic pathway activity, NTFD determines mass isotopomer distributions for all detected and labeled compounds. These data provide information on relative fluxes in a metabolic network. The graphical user interface allows users to import GC/MS data in netCDF format and export all information into a tab-separated format.

**Availability:** NTFD is C++- and Qt4-based, and it is freely available under an open-source license. Pre-compiled packages for the installation on Debian- and Redhat-based Linux distributions, as well as Windows operating systems, along with example data, are provided for download at http://ntfd.mit.edu/.

**Contact:** gregstep@mit.edu

## 1 INTRODUCTION

With the advent of systems biology, scientists now have the ability to probe and quantify the transcriptome, proteome and metabolome of virtually any organism (Fiehn *et al.*, 2000; Malmström *et al.*, 2007; Schena *et al.*, 1995). In the case of metabolic characterizations, gas chromatography (GC) coupled to mass spectrometry (MS) provides an excellent means of measuring and cataloging small molecule metabolites obtained from a variety of systems (Hiller *et al.*, 2009). Techniques such as GC/MS generate extremely rich datasets; however, these methods are somewhat limited in that they (i) require *a priori* knowledge of a given metabolite for identification; (ii) contain significant amounts of data not relevant to a given experiment; and (iii) provide a static view of metabolism. Indeed, such methodologies are often incapable of discovering new phenomena that have yet to be described in detail. Furthermore, quantification of metabolites can be of limited use when characterizing dynamic processes like metabolism. Metabolic fluxes provide the ultimate readout of *in vivo* enzyme activity (Stephanopoulos, 1999). To this end, researchers use metabolic flux analysis (MFA), which uses stable isotopes to generate information on the dynamics of metabolic processes (Sauer, 2006). As isotope-labeled substrates are metabolized and incorporated into downstream metabolites in an organism, the information contained in mass isotopomer distributions (MIDs) provides a readout on the relative fluxes in a metabolic network (Wiechert, 2001). Unfortunately, MFA requires comprehensive knowledge on the topography of metabolic networks in any organism under study. Therefore, as new organisms are identified and disease mechanisms are increasingly linked to aberrant metabolic processes, the need for discovery-based tools to detect and elucidate the dynamics of cellular metabolism is becoming apparent.

Here, we present a software, non-targeted tracer fate detection (NTFD), which detects all observable metabolites labeled by a stable isotope tracer within a GC/MS dataset (Hiller *et al.*, 2010). In addition, NTFD calculates MIDs for all ions derived from labeled compounds that are corrected for natural isotope abundance. As changes in intracellular reaction rates are directly reflected in the MIDs of metabolic intermediates, these data are crucial for the study of metabolic fluxes and enzyme activities. In contrast to MFA, NTFD requires no *a priori* information on the biological system (e.g. compound libraries or fragment formulas). Thus, NTFD provides a straightforward framework for combining the systems-level capabilities of traditional metabolomics approaches with the kinetic information that can only be ascertained through the use of isotopic tracers.

## 2 EXPERIMENTAL REQUIREMENTS

**Fig. 1.**
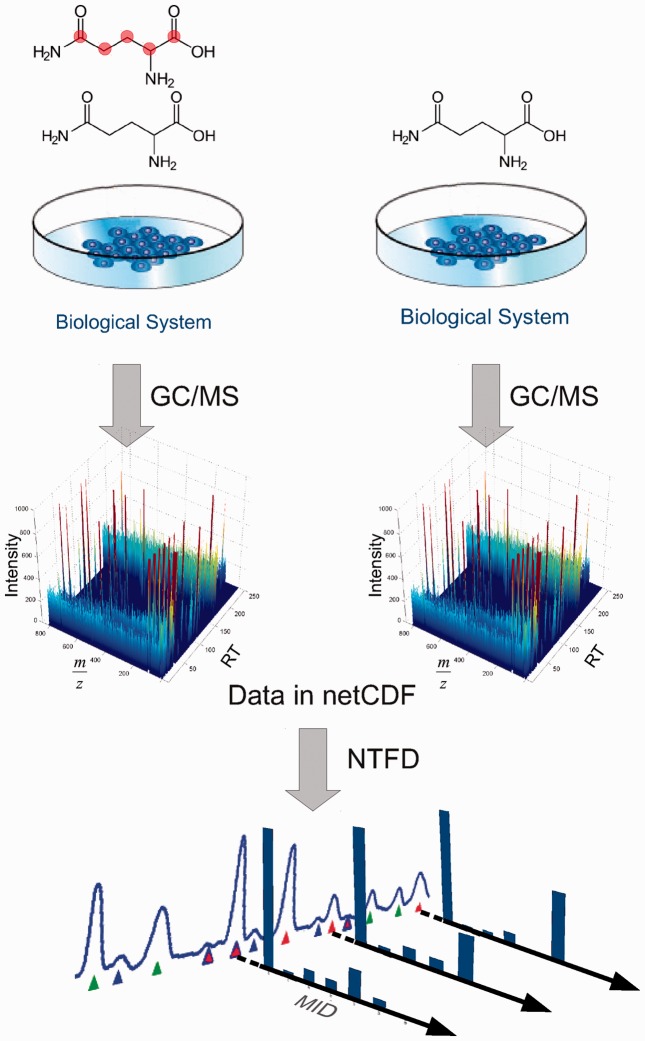
To detect the metabolic fate of a stable isotope tracer, two experiments must be performed in parallel under identical conditions: for the first experiment, the biological system needs to be incubated with a mixture of stable isotope-labeled tracer and unlabeled compound. For the second experiment, the system needs to be incubated under the same conditions with only unlabeled tracer

To generate tracer-specific isotopic enrichment patterns of metabolic intermediates, stable isotope-labeling experiments have to be performed using the tracer of interest (Fig. 1). NTFD works with nearly all stable isotopic-labeled compounds, including uniformly or singly labeled carbon, nitrogen, or sulfur tracers. In the case of deuterium, care must be taken, as the deuterium effect may cause labeled and unlabeled compounds to elute at different retention times from the GC (Turowski *et al.*, 2003). To reduce experimental noise, both experiments should be performed simultaneously and in replicates, which also enables NTFD to provide confidence intervals for each mass isotopomer. NTFD accepts mass spectrometric data in the common netCDF format, which is supported by nearly all current GC/MS instruments as an export function.

## 3 RESULTS AND FEATURES

The NTFD software performs all the following steps automatically to detect isotopically enriched compounds in GC/MS datasets:

- Ion chromatographic deconvolution and compound detection
- Compound pairing
- MID calculation by solving linear equation systems

NTFD applies an ion chromatographic deconvolution algorithm that we developed previously for the MetaboliteDetector software (Hiller *et al.*, 2009). Having detected all compounds present in the recorded mass spectrometric data, NTFD automatically pairs every compound detected in the labeled chromatogram to its counterpart in the unlabeled chromatogram based on retention time and mass spectral identity. This is the most critical step, because isotopic enrichment significantly changes the mass spectrum of a compound. As a typical GC/MS chromatograms contains mass spectra of >400 compounds, such a pairing cannot be done by manual inspection. To detect isotopically enriched metabolites, NTFD calculates the integrated difference spectrum for each compound pair (Hiller *et al.*, 2010). Every labeled ion fragment is characterized by a peak in the integrated difference spectrum, which is detected and evaluated by our software. Finally, the software calculates the MID for every detected and labeled fragment ion. Typically, the sum formula of the fragment ion is applied to calculate a correction matrix, which is needed to solve the linear equation system for accurate MID calculation (Lee *et al.*, 1991). Because of the non-targeted character of the NTFD analysis, such formulas are not available, and the software applies the mass spectrum of the paired unlabeled compound to set-up the correction matrix. For compounds with prevalent naturally occurring isotopes, such as carbon, a further correction needs to be applied. We have implemented an algorithm similar to the one proposed by Jennings and Matthews (2005). If replicates are provided, NTFD additionally calculates 95% confidence intervals and coefficients of determination to facilitate statistical evaluation of results. A full NTFD analysis can be performed on a standard computer in several minutes.

## 4 CONCLUSION

In conclusion, the NTFD software enables the detection of all stable isotope-labeled compounds derived from the applied tracer with a non-targeted approach. The C++- and Qt4-based software provides an easy-to-use graphical user interface and imports mass spectrometric data in the common netCDF format. Having detected all labeled compounds, MIDs for all compound ions are determined. If a reference spectrum library is provided, NTFD identifies as many compounds as possible. Finally, the results can be exported to a tab-separated file (tsv), which can be further processed with spreadsheet or statistics applications.

*Funding*: Supported by the Fonds National de la Recherche (FNR), Luxembourg (ATTRACT A10/03 & AFR 1328318) and the German Research Foundation (DFG) HI1400/1-1.

*Conflict of Interest*: none declared.
